# Mother and father depression symptoms and child emotional difficulties: a network model

**DOI:** 10.1192/j.eurpsy.2022.260

**Published:** 2022-09-01

**Authors:** A. Martin, D. Konac, B. Maughan, E. Barker

**Affiliations:** 1 King’s College London, Department Of Psychology, Institute Of Psychiatry, Psychology And Neuroscience, London, United Kingdom; 2 King’s College London, Sgdp, Institute Of Psychiatry, Psychology And Neuroscience, London, United Kingdom

**Keywords:** Parent-child, Mechanisms, Psychopathology, Internalising

## Abstract

**Introduction:**

Enhancing understanding of depression symptom interactions between parents and associations with subsequent child emotional difficulties will inform targeted treatment of depression to prevent transmission within families.

**Objectives:**

To use a network approach to identify ‘bridge’ symptoms that reinforce mother and father depression, and whether bridge symptoms, as well as other symptoms, impact subsequent child emotional difficulties.

**Methods:**

Symptoms were examined using two unregularized partial correlation network models. The study included 4,492 mother-father-child trios from a prospective, population-based cohort in the United Kingdom. Mother and father reports of depression symptoms were assessed when the child was twenty-one months old. Child emotional difficulties were reported by the mother at ages nine, eleven and thirteen years.

**Results:**

Bridge symptoms mutually reinforcing mother and father depression symptoms were feelings of guilt and self-harm ideation, whereas anhedonia acted as a bridge from the father to the mother, but not vice-versa (fig.1, network 1). The symptom of feelings of guilt in mothers was the only bridge symptom which directly associated with child emotional difficulties. Other symptoms that directly associated with child emotional difficulties were feeling overwhelmed for fathers and anhedonia, sadness, and panic in mothers (fig.1, network 2).

**Figure:**
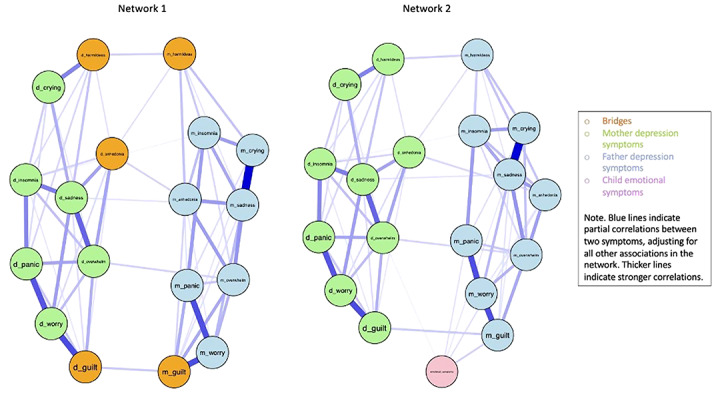

**Conclusions:**

Specific symptom interactions are central to the co-occurrence of depression symptoms between parents. Of interest, only one of the bridge symptoms associated with later child emotional difficulties. In addition, specific symptom-to-child outcomes were identified, suggesting that different symptoms in mothers and fathers are central for increased vulnerability in children.

**Disclosure:**

No significant relationships.

